# Electrically Modulated Multilevel Optical Chirality in GdFeCo Thin Films

**DOI:** 10.1021/acsaelm.4c01642

**Published:** 2024-12-16

**Authors:** Jun-Xiao Lin, Bo-Jun Chen, Shih-Min Hung, Wei-Hsiang Liao, Michel Hehn, Shih-Jye Sun, Yu-Ying Chang, Thomas Hauet, Julius Hohlfeld, Stéphane Mangin, Hua-Shu Hsu

**Affiliations:** ‡ Department of Applied Physics, National Pingtung University, No. 4−18, Minsheng Road, 90044 Pingtung, Taiwan; § Institut Jean Lamour, Université de Lorraine, CNRS, F-54000 Nancy, France; ⊥ Department of Applied Physics, National University of Kaohsiung, 700 Kaohsiung University Road, Nanzih District, 811 Kaohsiung, Taiwan

**Keywords:** optical chirality, circular dichroism, magnetooptical ellipticity, spin reorientation transition, GdFeCo

## Abstract

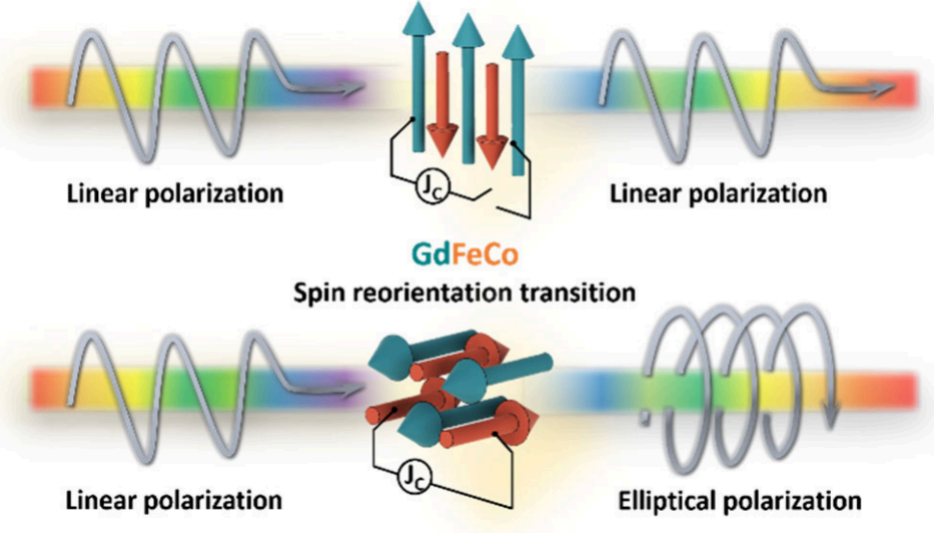

This study introduces a simple approach to dynamically control multilevel optical ellipticity in ferrimagnetic GdFeCo alloys by switching the spin orientation through Joule heating induced by electrical current, with the assistance of a low magnetic field of 3.5 mT. It is demonstrated that selecting specific compositions of Gd_*x*_(FeCo)_100–*x*_ alloys, with magnetic compensation temperatures near or above room temperature, allows for significant manipulation of the circular dichroism (CD) effect. This control enables the transformation of transmitted light from linearly polarized to elliptically polarized or the reversal of the rotation direction of elliptically polarized light across the photon energy range from visible (vis) to ultraviolet (UV). The efficacy of this method is rooted in the dominant contributions of FeCo to the CD effect in the vis-to-UV energy range. Because the magnetization of FeCo remains relatively independent of the temperature, substantial optical ellipticity is maintained for optical device applications, regardless of whether the compensation temperature is approached or crossed. Our results highlight the potential of GdFeCo thin films in chiral optics and demonstrate the selective contributions of rare-earth transition-metal elements to the CD effects, facilitating the design of advanced optical devices leveraging energy-resolved CD phenomena.

## Introduction

Optical chirality, also known as optical activity or optical rotation, refers to the property of certain materials to rotate the plane of polarization of linearly polarized light as it passes through them. In addition to optical rotation, chiral substances exhibit circular dichroism (CD), where they differentially absorb left- and right-circularly polarized light, leading to elliptically polarized light. Chiral substances are materials that lack mirror symmetry and thus have distinct left-handed and right-handed forms. Optical chirality is crucial in biochemistry and structural biology, such as revealing molecular structural information and determining the purity of chemical samples. On the other hand, it also plays a significant role in optoelectronics and photonics, where it is applied in the development of advanced materials and devices, such as optical isolators, modulators, and sensors.^1−5^ Recent advancements have introduced “chiral metamaterials”, composed of subwavelength metallic building blocks, showcasing potential in optical chirality.^6^ Fabricating chiral inorganic materials and revealing their unique quantum confinement-determined optical chiral responses are important methods in multidisciplinary research.^7^ Moreover, integrating chiral organic ligands into hybrid organic–inorganic perovskites offers a promising avenue for chiroptical materials.^8−10^ Nonetheless, current efforts predominantly focus on static chiroptical device development, leaving significant room for exploring dynamic chiroptical effects, especially through electrical modulation and large-area fabrication, aiming for efficient and ultrathin optical devices.^11−16^

Magnetic metal thin films generate optical chirality through magneto-optical (MO) effects, such as optical rotation and CD, which can be dynamically modulated by magnetic fields, electric fields, or currents.^17−20^ Although their poor transmittance limits some applications, magnetic thin films are particularly effective in scenarios where detecting or modulating changes in light properties is crucial. Recent advancements in quantum computation, information processing, and optical spin communication have generated renewed interest in magnetic thin films.^21−24^ The optical chirality induced by the asymmetry of spin magnetic moments in these films is of particular importance.

Among all types of magnetic materials, ferrimagnetic rare-earth transition-metal (RE-TM) alloy systems have been extensively studied for spintronic applications due to their easily tunable magnetic properties.^25−28^ Unlike pure ferromagnets, where TM elements solely contribute to magnetism, the RE sublattice in RE-TM alloys provides additional freedom to fine-tune the alloy’s magnetic properties.^29−32^ By varying the relative concentration or temperature, the magnetic anisotropy and dominant sublattice in these materials can be adjusted, exploiting the antiparallel coupling between the RE and TM sublattices when heavy RE elements are used. At fixed concentration, the so-called magnetic compensation temperature (*T*_comp_) corresponds to the temperature at which the opposing magnetic moments of the RE and TM sublattices are equal, resulting in a net magnetic moment of zero and a divergence of the coercive field.^30,31^ When the alloy’s temperature rises above the *T*_comp_, the dominant sublattice transitions from one element to another. Moreover, in the presence of an external magnetic field with appropriate amplitude, applied parallel to the original dominant sublattice direction, laser pulse induced transient heating of RE-TM alloys with *T*_comp_ < room temperature (*T*_room_) reverses the magnetization.^33^ These characteristics suggest a potential insight: optical polarization modulation might be achieved through electrically controlled heating and the application of a low magnetic field, making this material suitable for dynamically tunable optical chirality applications.

**Figure 1:**
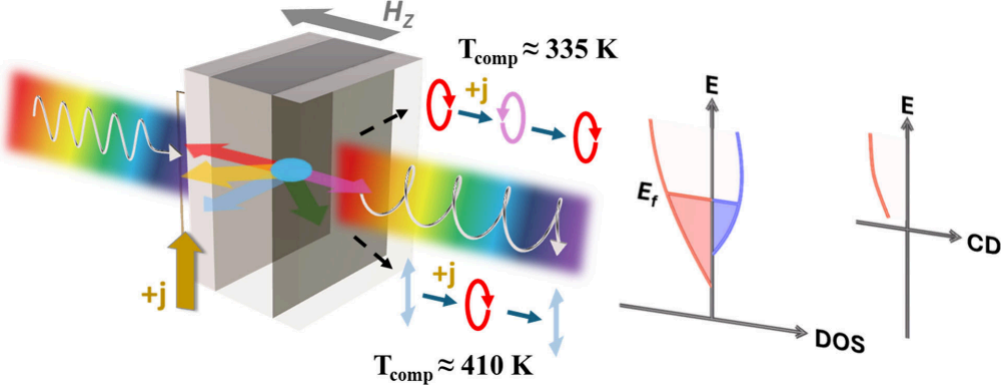
Conceptual schematic of electrical tuning of the optical ellipticity using Pt/Gd_*x*_(FeCo)_100–*x*_/Pt. When linearly polarized light, consisting of equal parts left circularly polarized (σ+) and right circularly polarized (σ−) components, passes through Gd_*x*_(FeCo)_100–*x*_, a magnetization-direction-dependent difference in absorption of these σ+- and σ–-polarized components induces CD, thereby altering the ellipticity of the transmitted light. A low magnetic field, oriented perpendicular to the substrate (*H*_Z_), is applied to enable heat to control the magnetization direction. For a sample with *T*_comp_ ≈ 410 K, the magnetization of GdFeCo gradually shifts from in-plane to out-of-plane as the current heats the sample, causing a CD effect and modifying the ellipticity of the transmitted light. Conversely, for a sample with *T*_comp_ ≈ 335 K, the magnetization may reverse due to current-induced heating across *T*_comp_, leading to a reversal in the direction of the optical ellipticity. This broadband CD effect originates from the band structure of the magnetic metal, which lacks a band gap.

In this study, we demonstrate that multilevel optical ellipticity states can be accessed and manipulated by controlling the magnetization orientation in ferrimagnetic gadolinium (Gd)–iron cobalt (FeCo) alloys through resistive heating by electrical currents of relatively low densities (in the range of 10^8^ A m^–2^) and the assistance of a modest applied magnetic field of 3.5 mT. GdFeCo is a RE-TM alloy with a tunable compensation temperature and exhibits soft magnetic properties, attributed to its weak local anisotropy.^34^ This weak anisotropy is a result of the significant s character and spherical symmetry of the Gd 4f electron cloud. These characteristics make GdFeCo an ideal model system for investigating the impact of electrical current on magnetic configurations when aided by an applied magnetic field. Figure 1 depicts a conceptual schematic illustrating tuning of the optical ellipticity through electrical current in a Pt/Gd_*x*_(FeCo)_100–*x*_/Pt system. We selected two concentrations with *T*_comp_ slightly above room temperature, around 410 and 335 K, respectively, and which exhibit, across *T*_comp_, magnetization rotation and magnetization switching, respectively. In both cases, we observed related and reversible changes in MO ellipticity across the photon energy range from visible (vis) to ultraviolet (UV). We show that a significant change in optical ellipticity, or even a sign reversal, can be obtained depending on the current-induced temperature rise and the difference between *T*_comp_ and *T*_room_. This substantial and tunable optical ellipticity, achieved through simple electrical current manipulation, offers new opportunities for dynamic control in chiral optics applications. It also underscores the importance of considering thermal effects in complex multisublattice magnetic systems for current-induced magnetization switching, particularly when the compensation temperature is only slightly above room temperature.

## Results and Discussion

### Characterization of the Compensation Temperature

**Figure 2:**
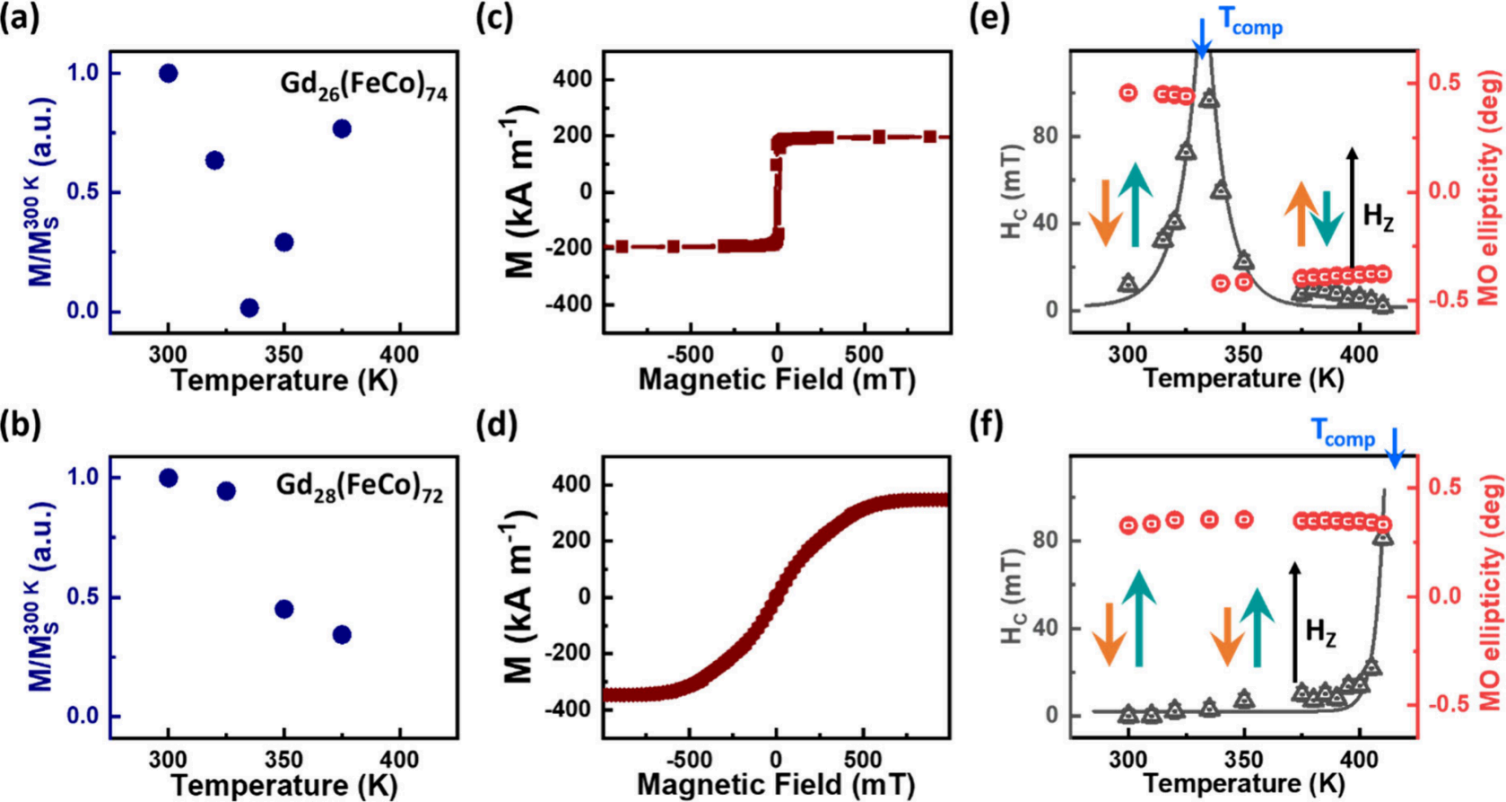
Quasi-static temperature-dependent magnetic properties of quartz/Ta (3 nm)/Pt (5 nm)/Gd_*x*_(FeCo)_100–*x*_ (20 nm)/Pt (5 nm) films for *x* = 26% and 28%. The temperature dependence of the normalized saturation magnetization (*M*/*M*_S_^300 K^) is shown for GdFeCo samples with (a) *x* = 26% and (b) *x* = 28%. Out-of-plane magnetization hysteresis loops measured at room temperature are presented for (c) *x* = 26% and (d) *x* = 28%. Temperature dependence of *H*_C_ and normalized MO ellipticity for the GdFeCo samples with (e) *x* = 26% and (f) *x* = 28%. Additionally, a schematic illustrating the variation in magnetization configuration is provided, showing the Gd magnetic moments (cyan arrows), TM FeCo magnetic moments (orange arrows), and magnetization directions relative to *H*_Z_.

Parts a and b of Figure 2 illustrate the relationship between the saturation magnetization (*M*_S_) and temperature for samples consisting of quartz/Ta (3 nm)/Pt (5 nm)/Gd_*x*_(FeCo)_100–*x*_ (20 nm)/Pt (5 nm), where *x* = 26% and 28%. These measurements were conducted using a commercial superconducting quantum interference device (SQUID), with an external magnetic field applied perpendicular to the substrate. Initially, both samples exhibit a decrease in *M*_S_ as temperature increases from 300 K. For the *x* = 26% sample, *M*_S_ reaches zero around 335 K, after which it begins to increase with temperature. The temperature at which the *M*_S_ becomes zero corresponds to the compensation temperature (*T*_comp_). On the other hand, the *M*_S_ of the *x* = 28% sample reduces monotonically with temperature within the measured temperature range (300–375 K), indicating a *T*_comp_ above 375 K. The out-of-plane hysteresis loops of the two GdFeCo films measured at *T*_room_ = 300 K, shown in Figure 2c,d, confirm that a closer proximity of *T*_comp_ to *T*_room_ leads to a lower *M*_S_ at *T*_room_. Moreover, the distinct easy and hard axis character of the loops indicates that the perpendicular magneto-crystalline anisotropy dominates the demagnetizing field for the *x* = 26% sample, but that the situation is reversed for the *x* = 28% sample where the high *M*_S_ leads to a large demagnetizing field. Thus, in the absence of an external magnetic field, the equilibrium magnetization is oriented along the perpendicular easy axis for the 26% sample but lies within the easy sample plane for the 28% sample.

Parts e and f of Figure 2 present the coercivity (*H*_C_) and normalized magnetooptical (MO) ellipticity signals as functions of temperature for samples with *x* = 26% and 28%, respectively. These measurements were taken using optical ellipticity spectroscopy with a photon energy of 1.55 eV. As expected, for the *x* = 26% sample, *H*_C_ diverges and the sign of MO ellipticity reveres around 335 K, corresponding to *T*_comp_. The point at which *M*_S_ in Figure 2a reaches zero at the compensation temperature while *H*_C_ diverges in Figure 2e is addressed in Supporting Information S1. On the other hand, for the *x* = 28% sample, *H*_C_ tends to diverge when the temperature exceeds 410 K, suggesting that *T*_comp_ is approximately 410 K. Note that our magnetic investigations revealed that at room temperature, the magnetization of the Gd sublattice is dominant over that of the Co sublattice in both samples. Supporting Information S2 shows the relationship between the field-dependent MO ellipticity signal and temperature measured for the two samples. The results demonstrate that the polarity of the MO ellipticity hysteresis loop for the *x* = 26% sample switches when the temperature rises above 335 K, indicating a change in dominant magnetization from the Gd sublattice to the Co sublattice. However, for the *x* = 28% sample, the Gd sublattice continues to dominate the net magnetization across the same temperature range, while the sample’s magnetic anisotropy gradually transitions from in-plane to out-of-plane.

Moreover, unlike the significant changes in *M*_S_ values observed in the SQUID measurements shown in Figures 2a,b, the absolute value of the saturated MO ellipticity does not exhibit a noticeable change (Figures 2e,f). It is generally assumed that MO effects, such as optical ellipticity, directly correlate with the *M*_S_ intensity in typical magnetic materials. This leads to the expectation that a significant decrease in *M*_S_ intensity would diminish the sample’s technical applications, such as MO recording. However, this expectation is not generally valid for ferrimagnetic RE-TM alloys for the following reasons.

First, the substantial changes in *M*_S_ observed as these alloys are heated toward *T*_comp_ arise from differences in the temperature dependencies of the RE and TM sublattice magnetizations, with the RE sublattice showing a stronger variation than the TM sublattice.^35^*M*_S_ represents the net magnetization, or the sum of the magnetic moments of Gd and FeCo, leading to a pronounced temperature-dependent change in *M*_S_. Second, the CD effect at a photon energy of 1.55 eV of these alloys within this compositional range is often predominantly influenced by optical transitions within the TMs, as is the case for GdFeCo.^32^ Consequently, the ellipticity does not vanish with *M*_S_ at *T*_comp_.

### Modulation of the Multilevel Optical Chirality through Electrical Currents

**Figure 3:**
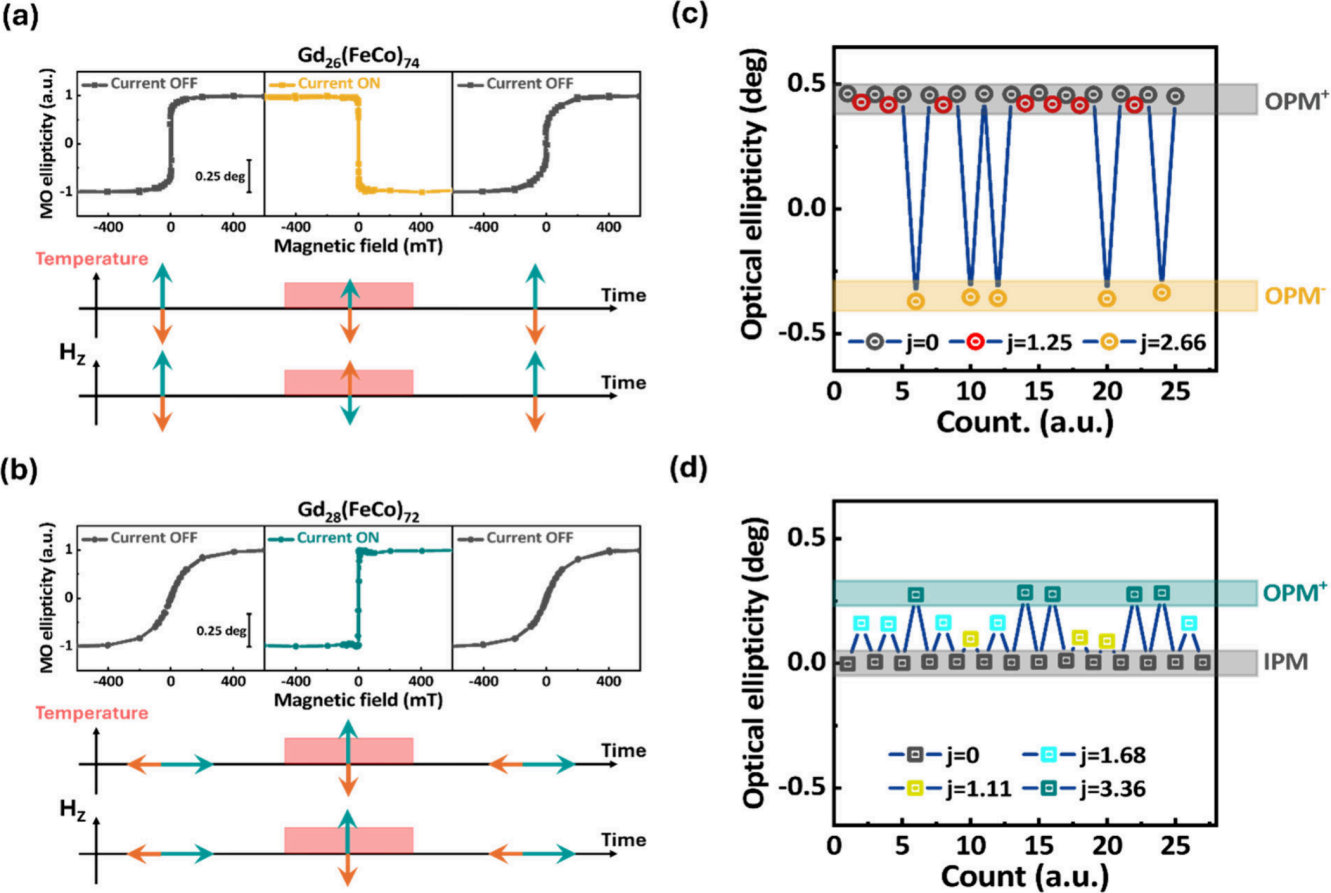
Electrical current control of optical ellipticity at a photon energy of *E* = 1.55 eV in quartz/Ta (3 nm)/Pt (5 nm)/Gd_*x*_(FeCo)_100–*x*_ (20 nm)/Pt (5 nm) films with *x* = 26% and 28%. Panels a and b show the on/off switching of the MO ellipticity hysteresis loop for the GdFeCo samples with *x* = 26% and 28%, respectively. The lower panels a and b provide schematics illustrating the changes in the magnetic configuration when the temperature is varied, and a DC magnetic field (*H*_Z_) is applied to the two samples. Panels c and d demonstrate the reversibility of optical ellipticity switching for the GdFeCo samples with *x* = 26% and 28%, respectively, under various applied electrical current densities (*j*, in units of 10^8^ A m^–2^) and an *H*_Z_ of 3.5 mT. The corresponding magnetization directions of the TMs are shown on the right: IPM represents magnetization in the plane direction, OPM^+^ denotes out-of-plane magnetization aligned with the initial direction, and OPM^–^ represents out-of-plane magnetization opposite to the initial direction.

Choosing a sample with *T*_comp_ slightly above room temperature allows for easy modulation of optical chirality, specifically optical ellipticity, through the heating effect induced by an electrical current. The detailed methodology for electrical current control of optical ellipticity is provided in the Experimental Section. Figure 3a demonstrates the process of current control of MO ellipticity hysteresis loops for the *x* = 26% sample. The initial state measurement shows an easy axis with a square-like shape, confirming that the magnetization is perpendicular to the film plane. Supporting Information S3 compares the hysteresis loops measured at room temperature using MO ellipticity at 1.55 eV with those obtained from SQUID measurements. While both measurements for the *x* = 26% sample exhibit behavior consistent with perpendicular magnetic anisotropy, differences in the magnetization behavior can be observed. These differences could indicate that the *M*_S_ measured by SQUID includes contributions from Gd sublattices, whereas the MO ellipticity primarily reflects the behavior of the FeCo sublattices. When an electrical current with a density of *j* = 2.66 × 10^8^ A m^–2^ is applied, the polarity of the MO ellipticity hysteresis loop reverses, indicating that the magnetization has switched direction. After turning off the current, the hysteresis loop returns to the initial out-of-plane magnetic configuration. For the *x* = 28% sample (Figure 3b), which has a higher *T*_comp_ than the *x* = 26% sample, the initial state exhibits a hard axis with neither MO ellipticity signal nor coercivity, suggesting that the magnetization lies in the plane direction, consistent with the out-of-plane magnetization (*M*/*M*_S_) hysteresis loop recorded at room temperature. Under electrical current application with the current density of *j* = 3.36 × 10^8^ A m^–2^, the MO ellipticity hysteresis loop turns into a square-like shape with a remanence close to one, confirming that the magnetization is now perpendicular to the film direction. Turning off the current restores the magnetization to the in-plane configuration. As discussed later, the lower panels in Figure 3a,b provide schematics illustrating the changes in magnetic configuration when temperature and a DC magnetic field are applied to the two samples.

To demonstrate tunability and reversibility, we applied varying current densities to control the optical ellipticity signal under an external magnetic field *H*_Z_ = 3.5 mT. This magnetic field strength was chosen because it is a typical residual value observed in electromagnet cores, and its amplitude is comparable to or greater than the *H*_C_ measured for the sample under the corresponding current densities. Parts c and d of Figure 3 present how different optical ellipticity states can be accessed using relatively low current densities for samples with *x* = 26% and 28%, respectively. By applying a sequence of currents with different amplitudes, we were able to manipulate the optical ellipticity. In the *x* = 26% sample, a sign reversal in the optical ellipticity signal occurs when *j* increases to 2.66 × 10^8^ A m^–2^. On the other hand, the *x* = 28% sample does not exhibit a sign reversal in the optical ellipticity; instead, it displays distinguishable multilevel optical ellipticity states that depend on the current amplitude. The optical ellipticity in both samples returns to the initial state after the current is turned off, confirming the reversibility of the effect.

### Broadband Optical Chirality Effect in GdFeCo Alloys

**Figure 4:**
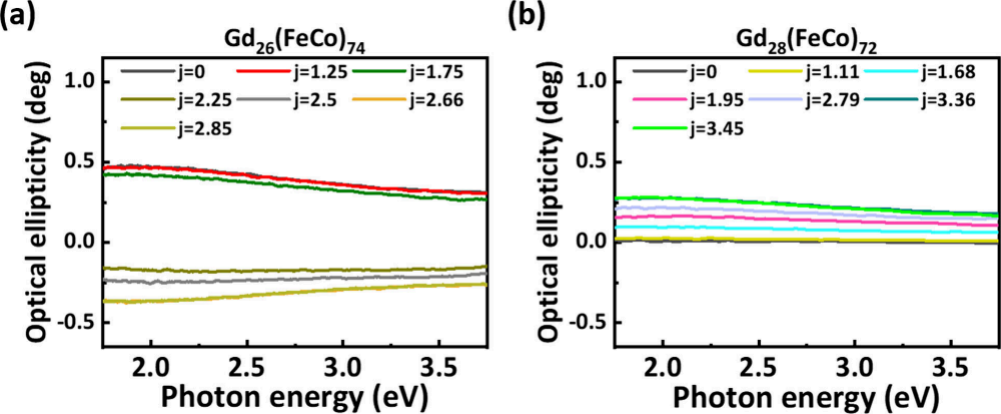
Photon energy-dependent optical ellipticity measurements for quartz/Ta (3 nm)/Pt (5 nm)/Gd_*x*_(FeCo)_100–*x*_ (20 nm)/Pt (5 nm) films with (a) *x* = 26% and (b) *x* = 28%, subjected to various applied current densities and measured in a 3.5 mT DC magnetic field. The current densities (*j*) are at 10^8^ A m^–2^ units.

After investigating the control of optical ellipticity at a specific photon energy, we now explore the changes in optical ellipticity under the application of electrical current at various photon energies using optical ellipticity spectroscopy. Parts a and b of Figure 4 illustrate the room-temperature current-manipulated optical ellipticity spectra of samples with *x* = 26% and 28%, respectively, under *H*_Z_ = 3.5 mT, with light energy excitation ranging from *E* = 1.75 to 3.75 eV. It is clearly observed that the entire spectrum reverses sign when the applied current density is high enough in the *x* = 26% sample, while the optical ellipticity intensity significantly increases with current density in the *x* = 28% sample. The ability to manipulate multilevel optical ellipticity states under different current densities, across the vis-to-UV photon energy range, is attributed to the band structure of GdFeCo.^32^ This band structure results in a joint density of states that varies only slightly with photon energy throughout the range of our MO ellipticity measurements. Typically, changes in the optical ellipticity are only observed at specific light energy excitation levels in chiral metamaterials, chiral perovskite, or semiconductor nanocrystals.^4,10,36^ The pronounced manipulation of optical ellipticity in GdFeCo over a wide spectrum highlights the potential for applying the optical chirality effect across different photon energies in a single material.

## Discussion

To elucidate the mechanism behind the current-controlled modulation of optical chirality described earlier, we have magnified the MO ellipticity hysteresis loops before and after current application in the low magnetic field region, as shown in Supporting Information S4. In the sample with *x* = 26%, we observe that when the current heats the sample to a critical level, reversal occurs even at a low magnetic field of 3.5 mT. This is because, when the temperature exceeds the *T*_comp_, the dominant sublattice magnetization shifts from Gd to Co. When the *H*_C_ is less than 3.5 mT, the Co magnetization direction aligns with the applied magnetic field, reversing the optical ellipticity (Figure 3a). This explains why optical ellipticity reversal can be achieved with such a low applied magnetic field. In the sample with *x* = 28%, we observed that current-induced heating triggers a transition in the magnetization direction from in-plane to out-of-plane upon reaching a critical current. This transition results from the fact that an increase in temperature toward *T*_comp_ significantly reduces the *M*_S_ while the magnetocrystalline anisotropy remains largely unaffected. Hence, the sample develops a weak effective perpendicular anisotropy, which enables the applied magnetic field of 3.5 mT to align the magnetization out-of-plane, thereby inducing a significant CD effect that alters the optical ellipticity (Figure 3b). The modulation of optical ellipticity occurs at multiple levels, driven by a carefully designed current-induced temperature rise, which controls the magnetization direction. Once the current is switched off, the heat dissipates, causing the sample’s temperature to return to room temperature, and the magnetization recovers to its initial state, as *H*_Z_ is comparable to or greater than *H*_C_. We demonstrated that by leveraging the different alignments of the RE and TM sublattices in response to the magnetic field, and the tunability of *T*_comp_ and *H*_C_, reversible control of optical ellipticity can be effectively achieved through the appropriate current-induced heating.

Moreover, Supporting Information S5 reveals that the optical ellipticity does not change sign when the polarity of the applied current is reversed. This observation suggests that the manipulations of optical ellipticity driven by current cannot be attributed to the conventional spin-transfer torque mechanism in the GdFeCo layer.^37^ As shown in Supporting Information S6, we estimated the temperatures reached by the samples during resistive heating. For the sample with *x* = 26%, the sign reversal of the MO ellipticity occurs when the current density exceeds *j* = 2.5 × 10^8^ A m^–2^, corresponding to a temperature above 335 K, indicating that the compensation temperature has been surpassed. In contrast, for the sample with *x* = 28%, the MO ellipticity becomes discernible when the current density exceeds *j* = 1.75 × 10^8^ A m^–2^, corresponding to an approximate temperature of 310 K. These findings align with the MO hysteresis loop presented in Supporting Information S2. To quantify the total power required for resistive heating in the observed MO ellipticity manipulation, we measured the sample resistance, which was approximately 28.5 ohms for the *x* = 26% sample and 69 ohms for the *x* = 28% sample. Using Joule’s law, *P* = *I*^2^*R*, we calculated the power required to drive the modulation of MO ellipticity. For the applied current range (0.055–0.17 A), the estimated power range was 0.086–0.82 W for the *x* = 26% sample and 0.21–2 W for the *x* = 28% sample. The significant alteration in magnetization direction under low current densities underscores the significance of thermal effects induced by electrical currents in complex magnetic systems with multiple sublattices, especially when the *T*_comp_ or spin reorientation transition temperature is slightly above room temperature.

Notably, the current-induced modulation of optical ellipticity in our study was achieved under a relatively modest external magnetic field of just 3.5 mT. This field strength was selected based on the remanence of an electromagnet, which does not require an additional power supply, and is comparable to the stray fields typically produced by conventional ferromagnetic materials like Co.^38^ Looking ahead, we envision that integrating the GdFeCo alloy with a Co layer could enable modulation of optical ellipticity by leveraging the exchange field induced by the Co, particularly when the heterostructure is heated above the *T*_comp_. In this scenario, the Co layer could effectively replace the external magnetic field used in this study. Eliminating the need for an external magnetic field could broaden the range of future applications in the field of optical chirality and make the modulation process more energy-efficient. In addition, there is the potential to achieve a delicate balance between thermal agitation and magnetic anisotropy by employing pulsed electrical current followed by rapid cooling, thereby establishing a facile method to control optical chirality.^39^ Although the observed ellipticity signal of approximately 0.5 deg (or around 1% differential absorption between left and right circularly polarized light) may not be the most prominent compared to other electrically controlled optical ellipticity values, it remains detectable even with less sophisticated setups, providing a moderate polarization contrast. This simplicity can be advantageous in specific applications, especially given the broadband nature of our method, which spans from the vis to UV range. This feature opens up possibilities for applications where such contrast is adequate, or where structural simplicity is prioritized, particularly in scenarios that require broad spectral coverage.

## Conclusion

This study demonstrates a simple method for dynamically controlling optical ellipticity using ferrimagnetic GdFeCo alloys. By selecting thin films with specific compositions that set their magnetic compensation temperatures near room temperature, we achieved precise control over the optical ellipticity with the aid of a low magnetic field of 3.5 mT. This low magnetic field could potentially be replaced by the stray field induced by an additional ferromagnetic layer in contact with the GdFeCo, enabling more energy-efficient control of optical chirality. Electrical heating induces spin reorientation transitions, thereby altering transmitted light from linearly polarized to elliptically polarized, or reversing the direction of elliptical polarization. By taking advantage of the different alignments of sublattices in response to the magnetic field, along with the tunability of compensation temperature and coercivity, it is demonstrated that reversible control and reversal of optical ellipticity can be effectively achieved through appropriate current-induced heating. The substantial change in optical ellipticity across the vis-to-UV photon energy range arises due to the absence of an energy band gap in the GdFeCo band structure. Our findings emphasize the selective contributions of RE-TM elements to CD effects within specific photon energy regions, offering insights for developing energy-resolved optical chirality devices.

## Experimental Section

To demonstrate the potential of GdFeCo alloy for chiral optical manipulation, we prepared multilayer structures with the composition Ta (3 nm)/Pt (5 nm)/Gd_*x*_(FeCo)_100–*x*_ (20 nm)/Pt (5 nm), varying the Gd concentration to *x* = 26% and 28%. The GdFeCo alloy used in this work contains a Fe-rich (FeCo) sublattice, with 90% Fe and 10% Co. For simplicity, we have consistently expressed the chemical formula as Gd_*x*_(FeCo)_100–*x*_ throughout this study. The heterostructures were fabricated using magnetron sputtering with a base pressure of 5 × 10^–8^ mbar. Before multilayer deposition, the substrate surface was etched with RF Ar plasma at 1 × 10^–2^ mbar for 5 min. The sample holder was rotated at several tens of revolutions per minute to ensure uniform layer thickness during deposition. The 20 nm Gd_*x*_(FeCo)_100–*x*_ layer was cosputtered from high-purity (99.99%) Gd, Fe, and Co targets, with composition control achieved by adjusting each target’s power and calibrated before deposition. From our previous research results with similar fabrication methods, the GdFeCo layer exhibits an amorphous structure.^29^

In this study, an in-plane direct current was injected into the heterostructure from the top Pt layer using the current in-plane geometry. The optical ellipticity of the GdFeCo layers was examined using a Jasco J-815 spectropolarimeter, with light incident normal to the sample plane. The light used has a fluence of 20 nJ cm^–2^, provided by a Xe lamp. Before measuring the optical ellipticity, the samples were initialized by applying an external magnetic saturation field (*H*) along the film plane (+*z* axis) and then turning it off. In our study, the low external magnetic field (3.5 mT) was generated by the remanence of an electromagnet. This finding indicates that even without additional power supplied to the electromagnet, the residual magnetic field from the magnetized core is sufficient to achieve optical ellipticity modulation in GdFeCo. The optical ellipticity signals were collected 10 s after the electrical current was applied, ensuring that all results were taken in a thermal equilibrium state. For all MO ellipticity measurements, an electromagnet with a field oriented perpendicular to the substrate plane (*z* axis) was used.

In the present work, we did not observe a significant diamagnetic contribution in the MO ellipticity measurements, due to the magnetic CD effect of the quartz substrate remaining minimal within the magnetic field range measured. However, when extracting the magnetization values from the SQUID measurements, we considered the diamagnetic contribution from the substrate. To obtain accurate magnetization values, we fitted a linear slope at the initial magnetization values at higher fields in the raw data, corresponding to the all diamagnetic background. This linear slope was then subtracted from the raw hysteresis loop data, resulting in the obtained data.

## Supporting Information Available

Relationship between net magnetization and coercivity, quasi-static magnetic field-dependent MO ellipticity, comparison of hysteresis loops measured via MO ellipticity at 1.55 eV and SQUID magnetometry, magnification of MO ellipticity hysteresis loops, optical ellipticity as a function of the electrical current density, and temperature distribution under varying current densities of quartz/Ta (3 nm)/Pt (5 nm)/Gd_*x*_(Fe_90_Co_10_)_100–*x*_ (20 nm)/Pt (5 nm) films for *x* = 26% and 28% (PDF)
