# Supplementary material for: Electrically Modulated Multilevel Optical Chirality in GdFeCo Thin Films
Source: ACS Appl Electron Mater. 2024 Dec 16;7(1):177–84. doi: 10.1021/acsaelm.4c01642 (PMC11736793; doi:10.1021/acsaelm.4c01642)
Supplement: Supplementary file 1 — el4c01642_si_001.pdf [file el4c01642_si_001.pdf]

Supporting Information

Electrically Modulated Multi-Level Optical Chirality in GdFeCo Thin Films

Jun-Xiao Lin[ab†], Bo-Jun Chen[a†], Shih-Min Hung[a], Wei-Hsiang Liao[a], Michel Hehn[b], Shih-Jye Sun[c], Yu-Ying Chang[a], Thomas Hauet[b], Julius Hohlfeld[b], Stéphane Mangin[b], and Hua-Shu Hsu[a]**

[a]Department of Applied Physics, National Pingtung University, No. 4-18, Minsheng Road, 90044 Pingtung, Taiwan

[b]Université de Lorraine, CNRS, Institut Jean Lamour, F-54000 Nancy, France

[c]Department of Applied Physics, National University of Kaohsiung, 700, Kaohsiung University Rd., Nanzih District, Kaohsiung 811, Taiwan

[*]Correspondence and requests for materials should be addressed to S. MANGIN (email: stephane.mangin@univ-lorraine.fr) and H.-S. HSU (email: hshsu@mail.nptu.edu.tw).

KEYWORDS: optical chirality; circular dichroism; magneto-optical ellipticity; spin reorientation transition; GdFeCo

S1. Relationship between net magnetization and coercivity in GdFeCo alloy

In Figures 2(a) and (e), M represents the net magnetization of the Gd and FeCo sublattices, defined as $M_{eff}= |M_{Gd} - M_{FeCo}|$. In many ferrimagnetic systems, there is a simplified relationship between coercivity (H_C) and M_{eff}, described by $H_C \approx \frac{2K}{\mu_0 M_{eff}}$, where K is the material's anisotropy and μ_0 is the vacuum magnetic permeability. As M_{eff} approaches zero, H_C rises sharply, leading to the observed divergence of H_C at the compensation.[1]

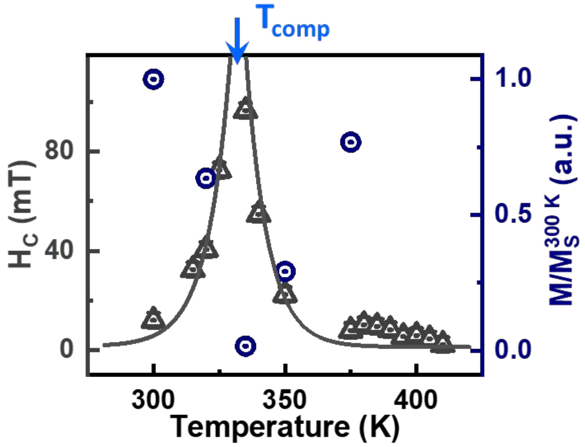

Figure S1. The temperature dependence of the coercive field (H_C) and normalized saturation magnetization ($M/M_S^{300\ K}$) for the $Gd_{26}(FeCo)_{74}$ sample.

S2. Magnetic field-dependent optical ellipticity for various temperatures

Figure S2 presents the relationship between the magnetic field-dependent magneto-optical (MO) ellipticity signal and temperature for the x=26% and x=28% $Gd_x(FeCo)_{100-x}$ samples. The magnetic fields (H_Z) were applied along the film plane (z axis). Note that our magnetic studies indicate that at room temperature, the Gd sublattice magnetization is larger than that of the Co sublattice in both samples. The results show that the polarity of the MO ellipticity hysteresis loop for both samples at room temperature is the same, confirming the dominance of the same magnetization sublattice. For the x=26% sample, the polarity reverses when the temperature increases above 335 K, indicating a change in dominant magnetization from the Gd sublattice to the Co sublattice. In contrast, for the x=28% sample, the Gd sublattice remains dominant across the entire temperature range, while the sample's net magnetization gradually transitions from in-plane to out-of-plane with increasing temperature.

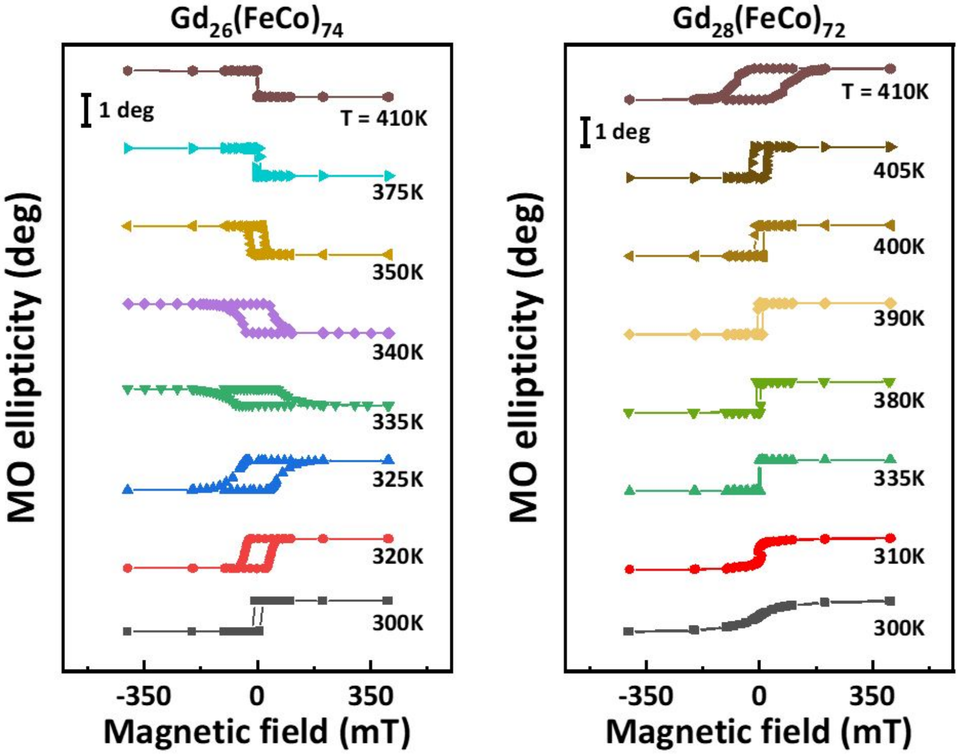

Figure S2. Quasi-static magnetic field-dependent magneto-optical (MO) ellipticity of Quartz/Ta (3 nm)/Pt (5 nm)/$Gd_x(FeCo)_{100-x}$ (20 nm)/Pt (5 nm) films for x=26% and 28%. The photon energy of 1.55 eV was used to measure the MO ellipticity. The data were extracted ten minutes after the measured temperature reached the set point.

S3. Comparison of hysteresis loops measured via MO ellipticity at 1.55 eV and SQUID magnetometry

Figure S3 compares the hysteresis loops obtained from MO ellipticity measurements at 1.55 eV with those measured using a SQUID magnetometer. Notable differences between the two methods were observed. The SQUID measurements capture the total magnetization contributions from both the Gd and FeCo sublattices, whereas the MO ellipticity predominantly reflects the magnetic response of the FeCo sublattices. This also highlights the limitations of relying on a single-energy light source in MO measurements to characterize the magnetic properties of materials, particularly in systems with multiple magnetic sublattices.[2]

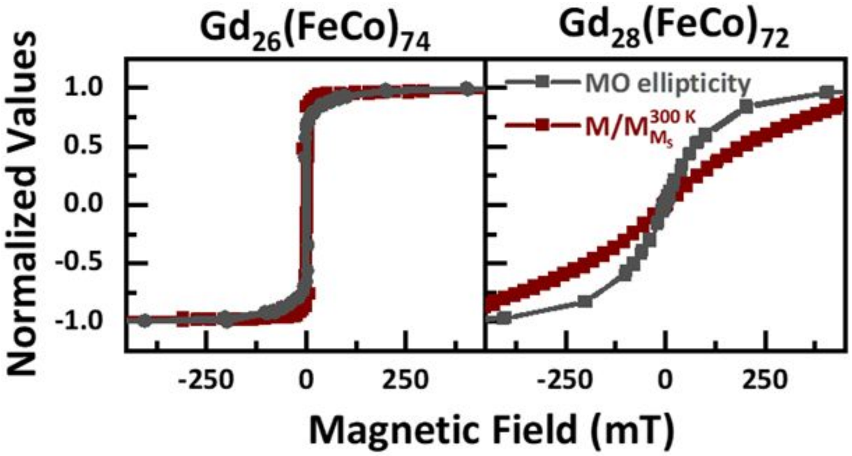

Figure S3. Comparison of hysteresis loops measured at room temperature using MO ellipticity at 1.55 eV and SQUID for x=26% and 28% samples.

S4. Magnification of MO ellipticity hysteresis loops

To confirm that the observed switching of the optical ellipticity is achieved with the assistance of a low field of 3.5 mT applied along the H_Z direction, Figure S4 provides a magnified view of the MO ellipticity hysteresis loops from Figures 3(a) and 3(b) in the low magnetic field region. For the x=26% sample, it is observed that the H_C decreases to approximately 1 mT when a current density of 2.66×10^8 Am^{-2} (Current ON) is used, but increases to around 5 mT at room temperature when no current is applied (Current OFF). In contrast, for the x=28% sample, the H_C is 2 mT at a current density of around j=3.36×10^8 Am^{-2} as the sample is heated, and it decreases to 0 mT when the current is switched off, where the magnetization lies in the in-plane direction.

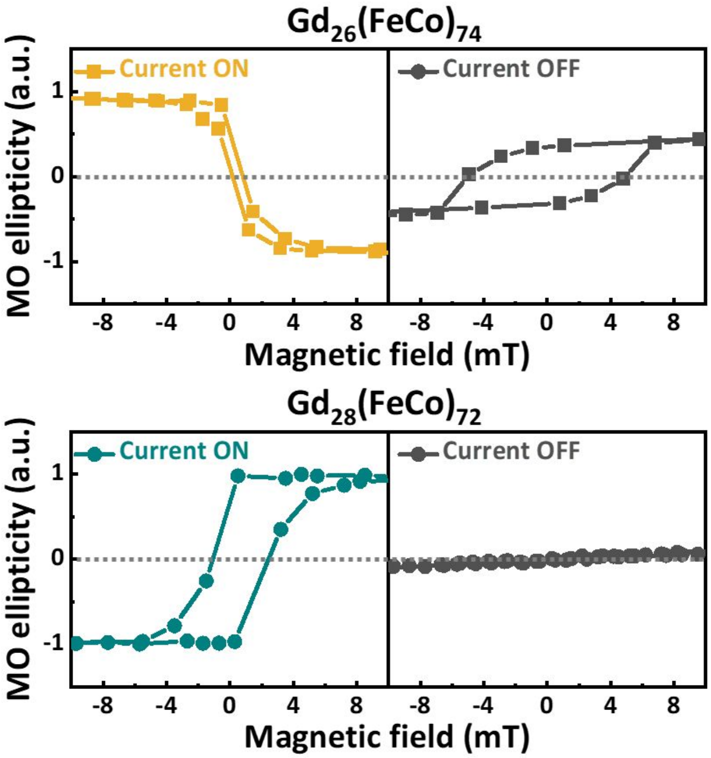

Figure S4. Electrical current control of optical ellipticity at a photon energy of E=1.55 eV in Quartz/Ta (3 nm)/Pt (5 nm)/$Gd_x(FeCo)_{100-x}$ (20 nm)/Pt (5 nm) films with x=26% and 28%. "Current ON" indicates that the sample is heated by the applied electrical current, while "Current OFF" signifies that the sample is at room temperature with no current applied.

S5. Optical ellipticity as a function of applied electrical current densities

Figure S5 shows the results of electrical current-manipulated optical ellipticity for the x=26% and x=28% GdFeCo alloys at room temperature. When no current is applied, the optical ellipticity signal is nearly zero for the x=26% sample, while it has a finite value for the x=28% sample. As the current is injected into the system, the optical ellipticity signal starts to change once the current density reaches a certain threshold. However, the sign of the optical ellipticity does not change with the polarity of the current for either sample. This result reaffirms that the manipulation of optical ellipticity in our work is due to the Joule heating effect. It is worth noting that although this effect is attributed to heating, it is pronounced and reversible.

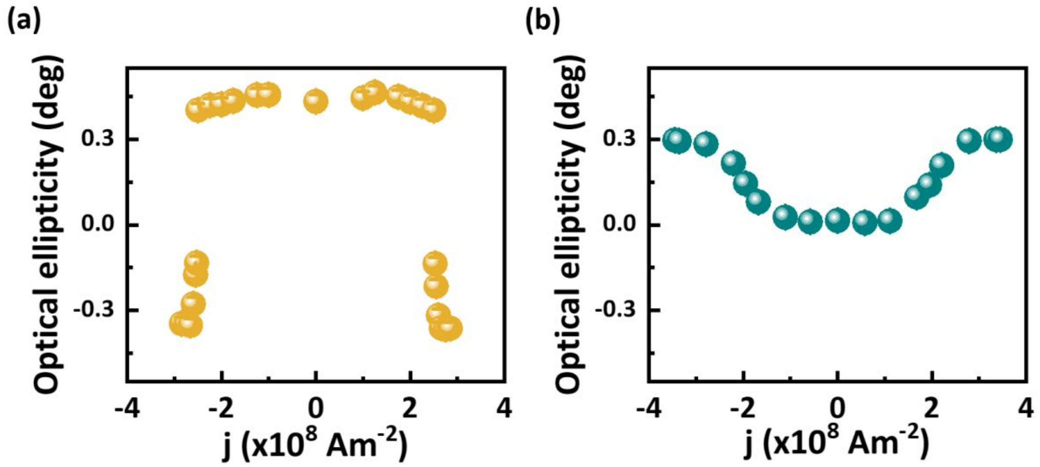

Figure S5. Optical ellipticity as a function of electrical current density for Quartz/Ta (3 nm)/Pt (5 nm)/$Gd_x(FeCo)_{100-x}$ (20 nm)/Pt (5 nm) films with (a) x=26% and (b) 28%. The optical ellipticity was measured under light excitation at an energy of 1.55 eV and an external magnetic field of 3.5 mT.

S6. Temperature distribution under varying current densities

Figure S6 shows the temperature rise measured using a FLIR C5 Compact Thermal Camera at varying current densities. As illustrated, the temperature increases by approximately 50 K within the range of applied current densities, with the exact rise depending on each specific current density. For the sample with x=26%, the sign reversal of the MO ellipticity occurs when the current density surpasses j=2.5×10^{8} Am^{-2}, corresponding to a temperature above 335 K. This indicates that the compensation temperature has been exceeded. This result aligns with the MO hysteresis loop shown in Figure S2, where the sign reversal is observed at the equilibrium temperature of 335 K. On the other hand, for the sample with x=28%, the MO ellipticity becomes apparent when the current density exceeds j=1.75×10^{8} Am^{-2}, corresponding to an approximate temperature of 310 K. This observation is also consistent with the MO hysteresis loop provided in Figure S2.

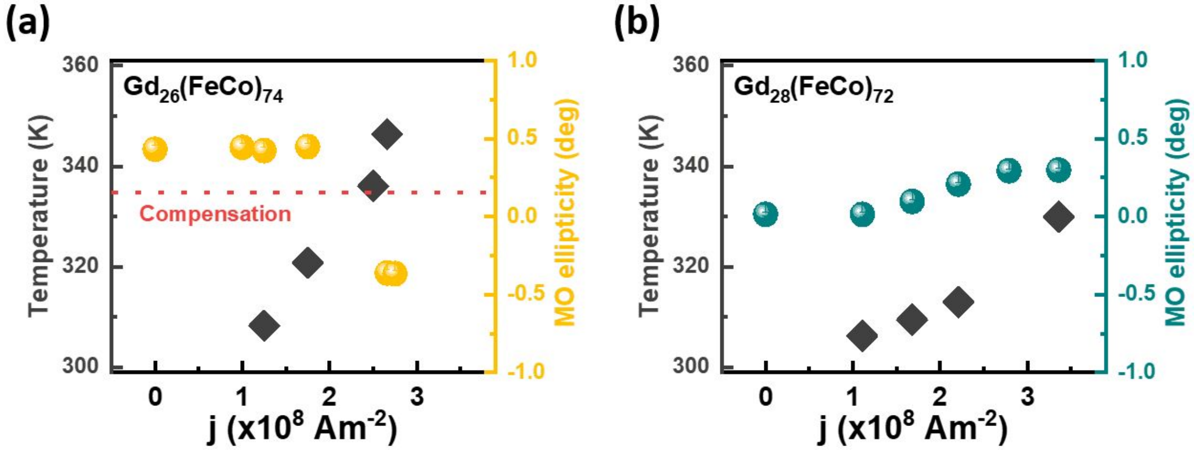

Figure S6. Temperature rises across varying current densities for (a) x=26% and (b) 28% samples.

REFERENCES

(1) Connell, G. A. N.; Bloomberg, D. S. Amorphous Rare-Earth Transition-Metal Alloys. *In Springer eBooks* **1985**, 739–752. https://doi.org/10.1007/978-1-4613-2513-0_60.

(2) Hu, C.-K.; Lin, J.-X.; Liu, H.-A.; Chang Chien, W.-H.; Wu, W.-B.; Lee, J.-S.; Lin, C.-R.; Mangin, S.; Chen, J.; Hsu, H.-S. Manipulation of energy-resolved magneto–optical effect in yttrium iron garnet films achieved by covering with nonmagnetic metals. *Chin. J. Phys.* **2024**, *90*, 717–725. https://doi.org/10.1016/j.cjph.2024.04.027.
